# Living in the era of codes: a reflection on China’s health code system

**DOI:** 10.1057/s41292-022-00290-8

**Published:** 2022-12-01

**Authors:** Haiqing Yu

**Affiliations:** RMIT University, Melbourne, Australia

**Keywords:** Health code, China, Grey digital divide, Elderly, Social credit system

## Abstract

This article offers a critical analysis of China’s health code system, a data-powered pandemic control and contact tracing system that supposedly subjects all individuals in the country to its panopticon control, a surveillance system that monitors and categorises the Chinese population into the healthy (green), the dubious (yellow), and the unhealthy (red). The article highlights the pretence of surveillance as care and the digital divide that normalises discrimination against the elderly and other digitally left-behind population. It also illustrates how, from policy making and technological design to user engagement, the health code system is implemented, optimised, and used in everyday life to meet the needs of the vulnerable population. The health code is better taken as a medium of adaptable and communicative process that can reset the relation between the system and the lifeworld. It is the process of interchange between the system and the lifeworld that deserves our critical attention.

A story entitled “the journey of yellow code and green code” circulated among my various WeChat groups in early August 2021. A plane flying from Xining (Qinghai) was forced to turn back from Lhasa (Tibet) due to severe weather and was instructed to land in Chengdu (Sichuan) and wait for the storms to pass. The announcement caused uproar among passengers, who declared “No, not Chengdu; we want to go back to Xining!” The reason was simple: stopping in Chengdu, a COVID red (high-risk) area, would turn everybody’s health code from green to yellow (if not red), which meant they would not be able to enter Tibet until their health codes turned green again (in 7–14 days). The captain offered a solution to the frustrated passengers: “if you do not turn on your mobile phones while waiting at the Chengdu airport, your health codes will not change colour, as the big data won’t have record of you being in Chengdu.” Everybody agreed. The plane was eventually instructed to return to Lhasa after half an hour at the Chengdu airport. Upon landing at the Lhasa airport, all passengers turned on their phones and their health codes remained green! Everybody was happy.

This story is a snapshot of the tyranny of the health code in China since February 2020 as China’s technological innovation in fighting the COVID-19 pandemic. The health code is a collaboration between tech companies and Chinese governments (of different levels) to serve as a COVID health status certificate and travel pass and tied with one’s national ID number. It uses GPS location and data mining technologies—accessible via Tencent’s WeChat^1^ and Alibaba’s Alipay, two of the most popular social commerce platforms in China—in combination with user self-reporting, to facilitate contact tracing, quarantine, and clinical management. It categorises people into one of three coloured QR (quick response) codes: green (low risk, free movement), yellow (medium risk, 7-day self-isolation), and red (high risk, 14-day mandatory quarantine). A golden colour is added to indicate one’s full vaccination status since May 2021—either on the rim of the QR code or as a logo in the middle of the QR code in most cases. The colour status system is used to regulate people’s movements, particularly to and from hotspots or any other restricted areas. It helps to monitor a de-personalised network of even fleeting contacts in places where social distancing is impossible, such as a bus or airplane. Its automated decision-making technologies track and identify one’s travel route and those of fellow travellers. If the colour code of one passenger changes from green to yellow, the colours of all other passengers’ health codes also change. 

China is not alone in using geolocation functions in mobile phones for contact tracing during the global pandemic. People all over the world are seeing radically different levels of surveillance through apps, services, and systems for contact tracing and quarantine management. In the race to contain the spread of COVID-19, national governments and technology companies have quickly produced and deployed automated contact tracing apps to tell users and public health officials whether somebody has potentially been exposed to the virus and whether a hotspot should be declared. As the list of such contact tracing apps compiled by MIT illustrates, some are temporary, voluntary, and lightweight (e.g., Australia’s COVIDsafe), while others are compulsory, pervasive, and invasive, such as China’s health code system (O’Neil et al. 2020).

Commentators outside China commonly hold the view that China’s health code and other contact tracing and behavioural management practices extend the regulatory mechanism of public health and human services authorities and open the door to unprecedented extension of biopower and patriarchal power over the social body. The *New York Times* calls China’s health code system “a template for new forms of automated social control that could persist long after the epidemic subsides” (Mozur et al. 2020). This contrasts with the prevailing argument in Chinese mainstream media that the health code and other contact tracing systems expand an individual’s capacity for self-protection and self-discipline in a time of uncertainty. Most Chinese seem to have accepted such a use of technologies as a state of exception.

In China, showing one’s health code and scanning venue QR codes are prerequisites for all public places, including public transport and shops. However, despite the high mobile internet penetration rate—over 60% of the population (of 1.4 billion) own one or more smart phones and over 99% of internet users access the internet via their smart phones—there are still many people without smart phones, mostly the elderly, the poor, and the disabled. They either cannot afford, or do not know how, or cannot operate, smart phones (and therefore the health code). These people are rendered immobile and subhuman by technology.

There are many stories about the impact of the health code on the elderly people. Media reports in 2020 have shown cases of them being denied access to social services (e.g., banks) and public transport for failing to pay their fees electronically or show their health codes. There are also viral videos and photos that are posted on Chinese social media like Weibo, such as: a video of an elderly woman sitting helplessly at a service counter in a bank for failing to renew her health insurance because she did not have a smart phone to show her green health code status or an e-wallet to make a payment; and a rural old man being kicked off a bus for not being able to show his health code to the bus driver, even though he had paid his bus fare. Such reports and accounts of health-code-entailed discrimination against the elderly have portrayed the moral paradox of the tyranny of the health code in a country where Confucian values on filial piety and care for the elders are supposedly held high. They have led to heated public discussions on the grey digital divide and subsequently public policy changes to address the problems.

This article offers a critical analysis of China’s health code system, a data-powered contact tracing system that is built on and part of Chinese digital governance. As a new mode of governmentality, it represents a communicative process in China’s adaptive authoritarianism (Chen 2010). The article adopts the Habermasian system vs lifeworld framework to examine the health code as a system to reorganising the lifeworld and its media-controlled subsystems. Lifeworld—defined as “socially integrated spheres of action,” often referring to culture and society as its two structural components—is distinct from the economic and administrative systems, which are the “objectified systemic networks” (Habermas 1987, p. 312) that constitute the “top-level design” (Tsai et al. 2021). The relations between “system” and “lifeworld” are relations of exchange and interchange. Habermas acknowledges that the functioning of objectified and formally organised systems depends on not only the internal ordering within the systems themselves but also the relation between the systems (mediated by money and power) and their environment (Habermas 1987). The “system” is hence not separate from but a sphere of society and “stands in a doubly contingent relation of interchange with other spheres of society” (Baxter 1987, p. 75).

The health code system, for example, is not a stand-alone or new system but depend on existing technological, political, legal, and cultural infrastructure and process; like technology itself, the process is accompanied with communicative actions by both the system and the lifeworld. Rather than simply a data-driven technological system to colonise the lifeworld, it is a medium of non-negotiable and yet adaptable and communicative process. In such a process, not everybody possesses the “structures of personality”—the skill, attitude, motivation and competences for speech and action “to calculate, to command, to organize, and to bargain” (Baxter 1987, p. 47). The elderly population, particularly those who are poor, illiterate, sick or disabled, belongs to such part of the society lacking in the structures of personality and ability. The call for adding “warmth” (human touch) to the health code system in the media (and social media) is an example of the interchange within the lifeworld and with the system.

The article firstly discusses the health code system in relation to China’s social credit system, a much-discussed digital surveillance system—known in the West as exemplary of the “surveillance state” and digital dystopianism (Strittmatter 2019)—to rate and rank individuals and gamify obedience by rewarding people of good character and behaviour and punishing people of bad behaviour and character. Like the social credit system, the health code system is a collective term for 33 or 34 sub-systems based on one’s provincial or municipal residence.^2^ It is used to rate and divide people into the *healthy and compliant* (green), the *dubious or unfortunate* (yellow), and the *unhealthy and non-compliant* (red). This discussion is based on document analysis and policy analysis. The documents are mostly policy documents issued by the Chinese central government and provincial governments and publicised on their official websites and/or circulated on social media platforms, all publicly available in relation to the discussion of the health code.

The article then investigates the technicalities and key features of the health code systems across the country via the walkthrough method. The walkthrough method allows researchers to engage with an app or platform in a systematic and immersive way, with a focus on the app’s technical and governance features, intended purpose, and embedded cultural meanings (Light et al., 2018). This is conducted via WeChat and Alipay personal accounts of the researcher and two China-based research assistants. Additional information is collected via WeChat groups. Many people in China discuss and often share their health codes in group chats on WeChat whenever there is a new wave of infections and lockdowns. Ten more different health codes are collected from the author’s WeChat groups. These health codes are posted by people over two years (February 2020-February 2022) from ten different provinces of China in a number of private group chats—from the author’s childhood friends’ group and alumni groups to research network groups (of Chinese colleagues). Permission has been sought from owners of the health codes to use them (anonymised) for research. In total twelve health codes are collected for comparison. It should be noted that the walkthrough method focuses on the technical features of the health codes and does not entail recruiting users as participants.

Finally, the article zooms in to examine the cultural politics of the health code as a data dividing device by focusing on the grey digital divide and how different stakeholders respond to the problem. It uses critical discourse analysis to analyse Chinese social media discussion of the problems that the elderly population has faced in face of the tyranny of the health code. The social media posts (such as the one described earlier) are accessed via Zhihu (Chinese equivalent of Quora), Weibo (Chinese equivalent of Twitter), WeChat, and Douyin (Chinese version of TikTok). The article does not intend to analyse individual posts—I have browsed these platforms on a regular (if not daily) basis and read tens of thousands of posts since February 2020—to gain insight on Chinese people’s views on the contact-tracing, automated decision-making technology, particularly on the discussions of the health code in relation to the older people. Such data is supplemented by 25 interviews conducted by two research assistants in Wuhan (10 interviews, 2021) and Chengdu (15 interviews, January–February 2022) for another but related project on digital kinship care and older people’s digital lives in the pandemic.^3^ People’s comments on the health code in their interviews—all anonymised and translated into English by the author—are selected for this article.

## Living in the era of codes: from the social credit to the health code system

Research on China’s health code has mostly drawn on surveillance studies scholarship and discussed its role as a state-sponsored surveillance infrastructure in consolidating the state’s centralising power in crisis management and normalising the expansive state surveillance (Cong 2021; Yang et al. 2021; Sun and Wang 2022). It has examined the role of digital platforms in conducting health surveillance and mediating state–citizen relations (Liang 2020). Although recognising the de-centralised, non-monolithic, and layered nature of the health code system, many scholars focus on the subjectification process of technological systems on society. From the social credit system to the health code system, technological systems and objects are regarded as bio-governance technologies to monitor, judge and regulate datafied bodies (Liang et al. 2018; Wu 2021a, b; Sun and Wang 2022); it is said to de-humanise governance and render citizens to a state of permanent visibility through social quantification (Liang 2020; Liang and Chen 2022). However, a closer examination of technological features and a review of literature suggest that a de-westernising approach to studying the health code system can shed new light on not only its technological affordances but also its embeddedness in the Chinese culture of social governance.

China has been featured in almost all reports on government-led automation systems. Its social credit system is widely discussed in the West as a digital surveillance system to punish people of bad behaviour and character and reward people of good character and behaviour. It is part of the global trend in the development and use of algorithms, computation, and data analytics that have already been used in the financial sector (e.g. banks and insurance companies) in the broader field of social governance. It is not entirely dissimilar to credit rating systems operating in other countries (Wong and Dobson 2019), but for its scale, scope and pervasiveness. Furthermore, there is no singular system that is controlled by the central government to rate each individual Chinese through its massive database. Instead, it is a decentralised mega project with multiple credit-rating systems, projects, and apps (Liu 2019; Schaefer 2019).

The “messy” understanding and interpretation of the social credit system (Ahmed 2019) is a result of the “chimera” nature of China’s allegedly high-tech social experiment with automated decision-making in governance—which in fact can be quite low-tech and de-centralised (Arsene 2019), as well as Western ideological bias toward digital China. It is also due to the tension between balancing the need for digitised services (or informatisation of government) on the one hand and concerns about privacy and surveillance on the other hand. Such messiness is further complicated by the increased state power aided by new technologies over population, including the profiling of people and targeting of policies and services (that is, to design policy to treat different profiles differently), and as such reduced citizen rights and equality. The current debate on China’s health code therefore should be examined, along with the social credit system, as part of the new global trend and symbolic system in resetting the relations between the “system” and the “lifeworld.”

A variety of surveillance technologies, contact-tracing apps and services are being experimented or implemented across the world with the aim of controlling the virus (Budd et al. 2020; Whitelaw et al. 2020). Many cities, such as London, used camera, sensors, robots, and AI algorithms to inforce social distance between people on the street or in the park. They have used health apps to track people’s location, record their travel history, quarantine status, and now COVID vaccination history to grant access to public facilities including public transport. China is one of the earliest countries in utilising digital technologies to tackle the pandemic. The Chinese governments at all levels and private companies have employed AI-powered surveillance cameras, drones, thermal cameras, and facial recognition to monitor and restrict the gathering of people in public during the outbreak of COVID-19 since early 2020. Mobile-phone and social-media-based applications are used to collect real-time data on people’s movement and allow health authorities to conduct contract tracing effectively and quickly. Machine learning algorithms are developed to detect and forecast COVID-19 cases across the provinces, identify regional transmission dynamics, and guide border checks and surveillance. At the local level, these detection and prediction models are used to guide clinical decision-making and resource allocation, such as identifying hospitals in need of critical care resources and medical supplies.

Apart from the high-level deployment of digital technologies, a contact tracing system has been implemented all over the country for people to record and check their movements and for authorities to track people’s travel history of the past 14 days. This QR-code system is called “Telecommunication Big Data Travel Card,” required for inter-city and inter-provincial travels in COVID-normal times and for all mobility access in COVID-lockdown times. Using mobile phone real-time location data, the contact tracing system was developed under the auspice of China Academy of Information and Communications Technologies (the Ministry of Industry and Information Technology) in collaboration of China’s three telecommunication operators: China Mobile, China Telecom, and China Unicom. It was rolled out in February 2020, the same month when the health code was developed.

The health code is built upon and connects with existing technological and social governance systems that are developed in the platformisation of cultural production and infrastructurisation of social governance in China (de Kloet et al. 2019). In developing and rolling out the health code system, private tech companies—despite professed reticence on handing over user data to the government prior to the COVID-19—have played the roles of harbingers, trainers, and collaborators of the centralised system of digital surveillance and biological governance. Before releasing the health code, Tencent and Alibaba, “had already developed real-time pandemic maps and apps to minimize exposure, conduct e-health queries, influence public opinion, and facilitate decision-making for critical medial resource allocation” (Chen et al. 2022, p. 2). The health code system is another public service mini app in the two tech giants’ super app ecosystems. Like the social credit system, it unfolds in a diversified manner and is composed of many localised subsystems. Also like the social credit system, it is not compulsory but enforced through soft coercion—required for access to public services and mobility in public spaces and transport. During the COVID-19 outbreak, the health code is incorporated into the social credit system to regulate social behaviours (Engelmann et al. 2019).

The successful adoption of the health code is not achievable without public trust in the system. In fact, the successful and appropriate use of any technology as an intervention will only be achievable when there is sufficient public trust and confidence in the technology and data (Bonsall et al. 2020). This has been demonstrated by a number of studies via surveys. These studies have shown that the majority of Chinese people support the health code system and trust the Chinese government (vis-a-vis private companies) in providing COVID information, protecting personal data, and implementing expansive surveillance systems (Min et al. 2020; Tang and Zou 2021; Wu 2021a, b; Liu and Graham 2021; Chen et al. 2022). Chinese citizens’ trust in their governments of all levels increased during the pandemic: a survey data shows that their trust in their national government increased to 98% in 2020 (compared to 95% in 2018) (Wu 2021a, b) and such trust is positively related to their support of precautionary practices in combating the COVID-19, including the continued use of the health code in post-pandemic times (Chen et al. 2022). Such a trust in the government and government-managed technological systems is cultivated and propagated in the state-controlled media and social media. Despite a growing awareness of privacy as individual rights, most Chinese people believe that public or collective interest outweighs personal privacy protection in terms of pandemic control (Liu and Graham, 2021; Liu 2021, 2022).

Such a positive attitude on the health code begs the question: what happens when things go wrong? Like the rolling out of the social credit system, the health code system has been peppered by glitches, loopholes, and breakdowns, particularly in 2020 when various local government raced to develop and implement their localised contact tracing systems to control the pandemic. The following analysis will attempt to answer the question by examining key features of various subsystems of the health code and the logics behind their optimisation.

## Walking into the health code

Several scholars have examined China’s health code via walkthrough analysis. This includes Sun and Wang (2022)’s detailed analysis of the Zhejiang health code, Meng et al. (2021)’s brief analysis of the Shenzhen/Guangzhou and Hangzhou/Zhejiang health codes, and Yang et al. (2021)’s quick walkthrough of the national health code on WeChat and Alipay. They have illustrated the step-by-step features of the mini apps, from registration and generating health report to their technological affordances and networked data management. This article will not walkthrough all the 12 sample health codes that the author has collected, which include Heilongjiang, Beijing, Shandong, Jiangsu, Shanghai, Zhejiang, Hubei, Sichuan, Guizhou, Fujian, Guangzhou, Hainan. Instead, it will summarise the key comparative features and highlight the add-on services and features of these health code mini apps. It will use the Sichuan health code for illustration.

As mentioned earlier, the health code system is composed of mini apps on WeChat and Alipay. It was pioneered in Shenzhen and Hangzhou by Tencent’s WeChat and Alibaba’s Alipay, in collaboration with local governments, and launched on 9 February 2020 and 11 February 2020 respectively, just days after Wuhan was in full lockdown from 23 January 2020. Alipay’s health code was developed upon “DingTalk employee health code”, a corporate internal system to monitor and manage its employees as they returned to work after the Chinese New Year national holidays (24 January-2 February 2020) to prevent COVID outbreak at the workplace. DingTalk is Alibaba’s enterprise communication and collaboration platform that quickly took off as China’s largest e-conference platform. Such a corporate initiative quickly evolved into a local government project at the request of the Yuhang District of Hangzhou (where the headquarter of Alibaba Group is located) on 4 February 2020 to Alibaba to develop a system for effectively managing residential compound lockdowns. The official health code system—known as Hangzhou (and later Zhejiang) health code—was a simplified version of the DingTalk employee health code. It used information reported by individuals and system-generated data from three dimensions—spatial (where one has been), temporal (how many times and how long one has been to a COVID hotspot), and relational (who are the close contacts)—to produce three coloured codes: green, yellow and red.

**Fig. 1:**
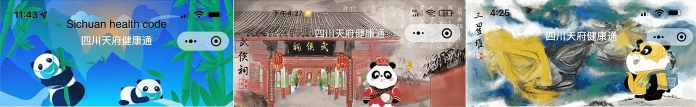
Vaccination status: unvaccinated or not fully vaccinated (mask-on panda, left) vs fully vaccinated (mask-off pandas, middle and right); dynamic backgrounds vary from person to person

The WeChat health code followed a similar corporate-to-public pathway, from Tencent’s headquarter in Shenzhen to other parts of the Guangdong province. Other cities and provinces quicly followed suit in rolling out their own local health code systems in collaboration with either Alipay or WeChat. The health code mini apps on WeChat and Alipay now covers all 33 administrative regions in Mainland China. Since May 2021, a golden colour is added to indicate one’s vaccination status, as a golden rim, a golden mascot in the middle, or a golden tag of “fully vaccinated” under or next to the user’s name. Some health codes incorporate additional and local features, like the panda mascot and iconic Sichuan scenic attractions as background in the Sichuan health code, to indicate users’ vaccination status (Fig. 1).

**Fig. 2:**
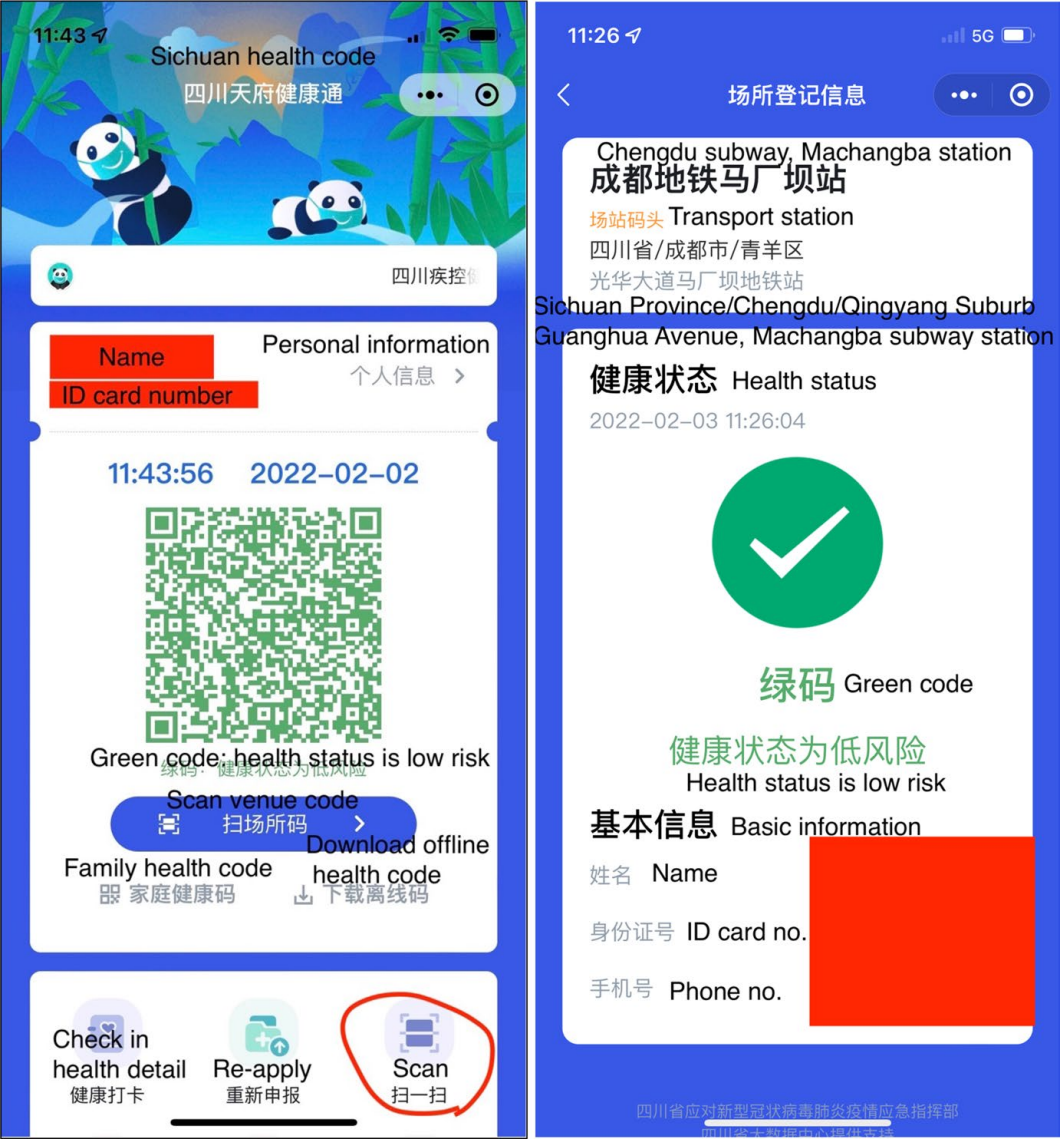
Sichuan health code: WeChat interface (left) and real-life application when using the “Scan” function (right)

All the different health codes follow the same algorithmic logic in collecting data and generating the three-coloured QR codes. They are all dynamic with automatic refreshing functions to prevent fraud and protect privacy. This means screenshots of any health code will not work. Showing a green health code is prerequisite for mobility, from getting out of one’s residential compound to taking a bus or train or airplane. For contact tracing, scanning venue QR codes via the health code function is an essential entry requirement in most public places such as subways, supermarkets, schools, hospitals, and government agencies (Fig. 2).

Like the social credit system, the health code system was rolled out in an uneven, fragmented, and “messy” manner in its early stage. The most prominent problem was related to the data walls among and opaque algorithms behind multiple health code systems at provincial and municipal levels. Many people went to social media to vent their frustration, with some complaining about “one person, multiple codes” and others about algorithmic arbitrary decisions in changing the colour of one’s health code (e.g. changing from green to red even when one had not left one’s home for over two weeks). The “one person, multiple codes” problem was caused by data walls among different health code apps, which required people to download and register a new app when they travelled from one region to another. The Chinese government responded to these complaints. On 29 February 2020 a national health code called “Epidemic Prevention Health Code” was launched on the website (www.gjzwfw.gov.cn) and app of the General Office of the State Council. Then the data interoperation, mutual recognition, and integration process between the national and local health code systems was mandated, requiring all local health code systems to share data and integrate into their administrative regions and national systems (accessible via WeChat and Alipay) by the Chinese New Year (12 February 2021) to enable “free movement with one code.” The process was not smooth, with some regions slow to enact the data-sharing and data-interoperation directives from Beijing. Inter-provincial travellers could find their health codes turning from green to yellow (7-day quarantine upon arrival and another 7-day quarantine upon returning) when they travelled to a different administrative region that had not completed the integration process. Such a problem has been less heard of since mid 2021, according to interviewees.

**Fig. 3:**
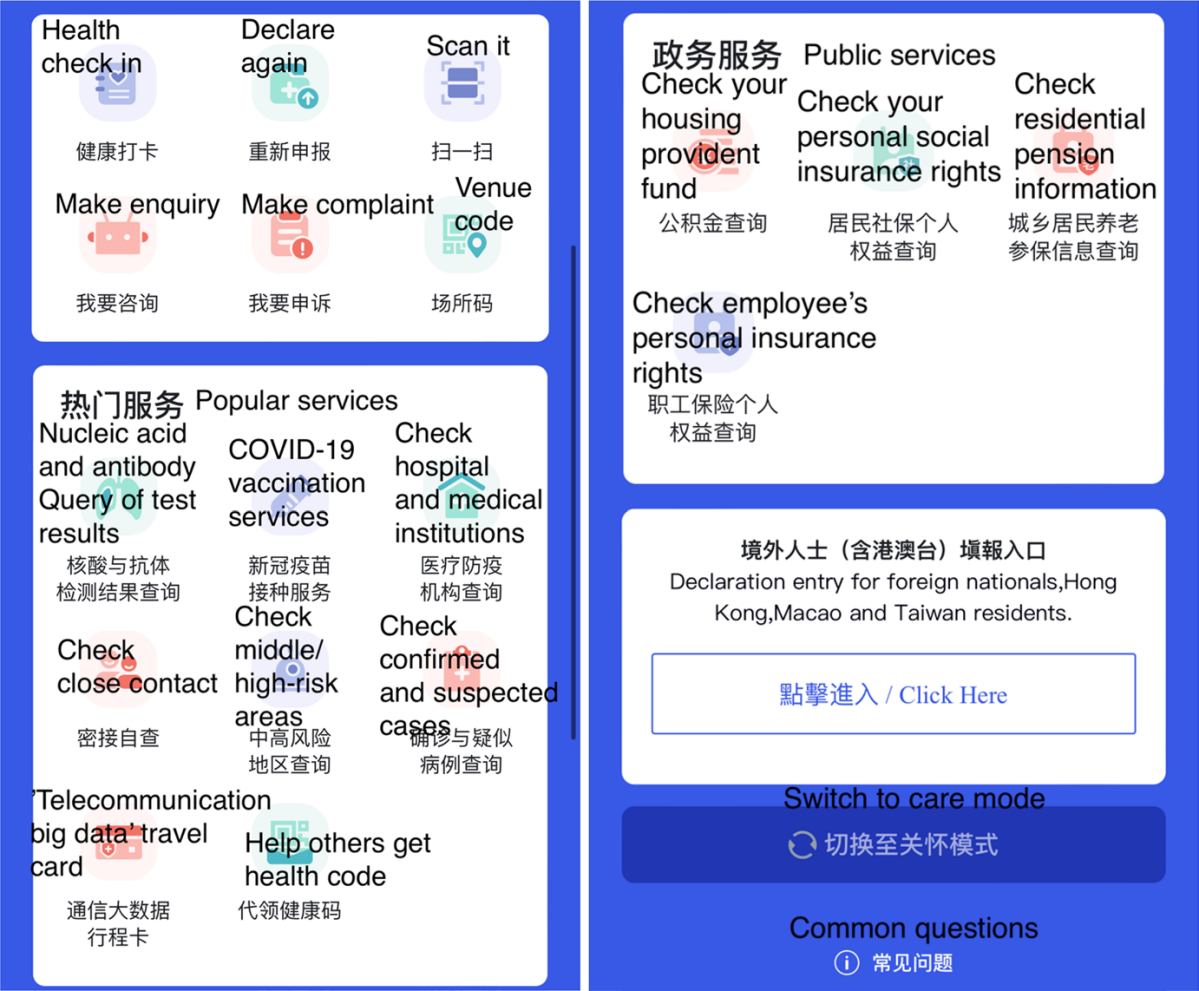
Four sections of key services on the Sichuan health code

The data interoperation and sharing between the 33 local health codes and the national health code only covers the basic information. In reality, the “one code” (the national health code) is less popular than the local health codes, which are no longer simply a technology for contract tracing and human mobility surveillance but a new portal for a wide range of public health services related to the pandemic control. The Sichuan health code, for example, has three major sections of functions in addition to the key colour status section (Fig. 3). The first section is the most standard one, with data integrated with the national health code system. It has six functions: “health check in,” “declare again (repeal),” “scan,” “enquiry,” “complaint,” and “venue code.” Most people only use functions of this section. The second section, “popular services,” includes eight functions that are related to testing, vaccination, and information on local pandemic control; the “help others get health codes” function (see below) is in this section. The third section, “public services,” is not directly related to COVID-19 but functions as a public service platform for social welfare services. There is a fourth section for foreigners (including residents of Hong Kong, Macao, and Taiwan) to register in the health code system. It provides the basic services and functions (as in the first section).

What needs to highlight are the “family health code,” “offline health code,” and “help others get health code” functions in the health code (Figs. 2, 3). These are add-on functions following a public outcry at the technological tyranny over and systemic discrimination against the vulnerable populations (e.g. the elderly and disabled). This will be further discussed in the next section.

## Living with the health code: the “remainder life” paradox

As mentioned earlier in the article, there have been many cases of older people being denied public transport for failing to show their health code or denied entrance to shops, hospitals, and banks for not being able to scan the venue codes. There are many posts on Weibo, Zhihu, and WeChat to express people’s frustration and outrage at the “cold” and “exclusive” nature of the health code. They express the feeling of “helplessness under the control of machines and big data” when the health code dictates one’s mobility and length of quarantine (https://zhuanlan.zhihu.com/p/107085476), the shared frustration that “fighting with technologies adds another level of stress when it’s already hard (for us) to fight the virus” (quoted from the author’s WeChat groups), or seniors’ sense of helplessness when “outside a building but unable to enter it” (interview). The health code tyranny is especially impacted on the older people (over 70 years old) who live alone and without family.

China has become an aging society, with 12.6% of its population or 176.03 million aged 65 or above by the end of 2019, a rise from 6.96% in 2000 (Huang et al. 2020). COVID-19 has forced a large number of senior citizens to adopt the prevalent digital lifestyle in China. In 2020 there was a further 14.2% increase rate in their adoption of the internet, mostly among the younger cohort of the elderly population (in their 60 s and early 70 s) who are driving the growth of China’s silver economy.^4^ However, with a 41.8% internet adoption rate among the elders, the majority of them are still left behind in China’s digital great leap forward (CNNIC 2021). The majority of non-internet users are in rural areas, which has a 62.7% internet penetration rate; metropolitan centres like Beijing and Shanghai have over 90% internet penetration rates (ibid). These non-netizen elderly people are regarded as the “remainder life” (*yushu shengming*) (Wu 2020), the mere life that cannot be digitised, coded, and “normalised” in the era of digital biopower. They are the technologically left-behind or left-out population, the technology and information have-less in the era of prevalent digital connectivity, information explosion, and attention economy. In the new normal of digital surveillance via health code during COVID-19, the remainder-life elderlies are denied of “intelligent” services in daily life such as travel, consumption, medical treatment, and shopping.

Senior people are not a homogenous group and different in adopting technology-related skills. Many of them refuse to be “digital refugees,” a term used in China to refer to the “remainder life” individuals vis-à-vis the digital natives and digital migrants^5^; they have learnt to use mobile phone apps—particularly WeChat—to communicate, socialise, seek information, use digital payment to pay bills and book tickets, and to access health code. But most of the older cohorts (over 75 s) are still at best partially digital users: they may use smart phones (often hand-me-down phones from children and grandchildren) and can use WeChat to communicate with family members and friends or watch Douyin videos; but they do not know how to use health code or scan QR codes via WeChat or Alipay in order to enter public places or take public transport. They also struggle with more advanced digital solutions in e-commerce, mobility, welfare, and social services that are put in place for the digital natives. Because of their age-related health problems, many old people cannot read on small screens, control their fingers, or remember simple instructions.

According to the CNNIC (2021) survey data, 27.2% of non-netizens believed that their inability to enter or exit public places due to the lack of health codes was ranked first, followed by the inability to make cash payment (25.8%) and to buy tickets or handle registration (24.9%). It is important to note that 24.6% of surveyed participants who are non-Internet users reported having difficulty in accessing social services due to the reduction or closedown of offline service outlets. This leaves those “remainder life” sections of the population who do not possess a smart phone or know how to use it, including many elderlies and people of lower socioeconomic status, at a distinct disadvantage. As Li (2020) writes for Sixth Tone, “Given how vital the internet was at the peak of the epidemic, when it was the primary means of doing everything from buying groceries to socialising, those unused to the technology faced an elevated risk of food shortages and potential difficulties in getting medical assistance.”

People call for social services to provide “warmth” and humane touch to accommodate the needs of the vulnerable and digitally left-behind population, such as enabling face-to-face staffed service and identity-card check-in options for those without smartphones or unable to scan QR codes, in addition to the self-help e-services. In response to such public outcry, in late 2020 the State Council issued notices to public service institutions and private businesses that they cannot refuse cash; that scanning the health code must not be the only way to enter a building; that ID cards (already linked to the national health code system) can be used by authorised persons to check and track one’s health status; and that hospitals must retain a proportion of on-site and telephone appointments as well as human-to-human service windows. At the same time, “mobile phones 101” courses by local governments and community-based services and educational programs (mostly run by neighbourhood committees and local libraries, staffed by volunteers) are rolled out to help the elderly population to order groceries online and teach them basic skills in digital and mobile communication, such as the use of the health code on their phones.

In response to the public outcry, Chinese tech companies developed tailored content, apps, and designs for the elderly and less tech-savvy demographics. These include: family-sharing features (on Alipay and WeChat) to allow children to pay for products or access the health code on behalf of their parents; lite version and/or senior mode with larger font size and simplified interface with voice commands and screen reader features for the visually impaired—known as the “care mode” in the health code mini-apps; and short-form videos to guide seniors to use their apps and how to access basic services on their apps (SCMP Research 2021). Other elderly-friendly modifications are also added to the health code. The family-sharing features, known as “family health code” in the Sichuan health code (Fig. 2, below the green QR code), allow users to apply for and scan health codes for junior and senior family members. Maximum five family members can be added. Apart from family health code, there is also an option for users to download their health code for offline use, valid for a week (also see Fig. 2). It is popular among users, mostly budget-conscious elderly, who do not have data plans on their mobile phones, to have access to public places. Instead of scanning venue codes, people can present their offline health code for venue staff to scan for verification of their COVID health status.

For people who do not have smart phones or are not with family members, the Sichuan health code enables a proxy service called “help others get health code” (Fig. 3). While the family health code function allows one to get family members’ health codes, the “help others get health code” function enables a non-family individual to have access to another person’s health code by inputting the person’s ID number. This function is often used in public venues and on public transport when entry staff or fellow travellers would use this function to help those in need. My interviewees have reported that most public venues have staff and volunteers to help people access the health code and scan QR codes.^6^ There have been many accounts on Chinese social media and from my interviewees about their or friends’ experiences of helping older people access their health codes. One interview recounts a story of her encounter in late 2021 with an old man being refused by the bus driver for not having any means to verify his health code status. I use this story to illustrate how the technology has evolved and optimised to meet real-life challenges and how ordinary people have responded to the system by adding human touch and warmth.

“It is so hard for both the old man and the bus driver,” the young woman in her 20 s said, “as the bus driver sobbed after forcing the old man off the bus and the old man was almost in tears too.” She continued to say,

We all know that the bus driver must comply with the government’s COVID requirement for services or risked losing his job. We don’t blame him for being rigid (with the rules) or rude (to the old man). No one is easy. So I stepped out to help the old man to access his health code on my phone (by using the “help others get health code” function). It took a couple of minutes, so the whole bus had to wait. Some passengers complained (for the delay) but most people were sympathetic. The old man thanked me profusely. He sat beside me and held my hand until I had to get off the bus. I can still feel the warmth of his hand.

## Conclusion

The story at the beginning of the article contrasts with the story above: one illustrates the coldness or heartless of China’s health code system and its utilitarian nature in the name of national and public interest; the other illustrates the progress in China’s technological response to the pandemic control and the human dimension or warmth in response to the tyranny of the technological system. The health code system is a data-driven experiment in China’s digital governance. It is among many other codes and systems that are developed by private technology companies in collaboration and partnership with Chinese governments at different levels. Together with the social credit system, the health code system is believed to represent a new mode of digital authoritarianism that challenges the Western liberal system and multistakeholder model of Internet and social governance (Wang 2021; Dragu and Lupu 2021; Khalil, 2020). Such a new mode of digital authoritarianism, as this article has illustrated, is situated in the Chinese political, technological, and cultural systems. It is networked, adaptive and responsive (MacKinnon 2011; Heurlin 2016). The system is also innovative and collaborative in meeting real-life problems of the lifeworld, from its technological design and optimisation to its incorporation into the public policy infrastructure and to its adoption by the Chinese people.

The health code has achieved what other codes and systems fail to attain—it is the only national system that is installed on the smart phones of over 70% of the Chinese population, that is, 986 million people (99.7% of China’s 989 million netizens) by the end of 2020 (CNNIC 2021); and the number has continued to grow. It is “more effective than the ID card system and more pervasive than the Skynet system”—what a “magic weapon,” commented by a netizen sarcastically on Zhihu in May 2020.^7^ The concern that the health code system can be “used against less legitimate targets than COVID-19” in the future (Courtney 2020) is shared by people in and outside China. This concern is legitimate given the Party-state’s track record in using digital, automated-decision making technologies to strengthen its governance over the datafied social bodies. Like many countries in the world, China becomes an “infrastructural state,” as digital platforms and technologies are now playing the role of basic service infrastructure and public utility (Bach 2016; Chen and Qiu 2019). The ownership and governance of these platforms and technologies are pivotal in the tension between national or public *interests* and individual digital and human *rights* in the era of codes.

The “magic weapon” of the health code system is a double-edged sword: one is either subject to its panopticon surveillance and hence “protection,” or excluded from enjoying basic services and citizen rights. As Szreter and Breckenridge (2012) have argued, it is often those who have been excluded from the gaze of the biopolitical state that have suffered the worst consequences, being unable to access essential services such as healthcare. “Living in the era of codes” is used to summarise such a conundrum in the COVID new normal in China, where people are probably the most “coded” in the world. This article has highlighted the pretence of surveillance as care and the grey digital divide that normalises discrimination against the elderly and other digitally left-behind population. It has also illustrated the operation of China’s new mode of governance through an analysis of the health code system—the way it is developed, rolled out, and optimised in response to the pandemic-control challenges and real-life needs of the people. Such a mode is realised through policy making, technological design, and user engagement (among one another and with technology). The health code is better taken as a medium of adaptable and communicative process that can reset the relation between the system and the lifeworld and through which the interchange between the system and the lifeworld may create a new symbolic system via “authoritarian participatory persuasion 2.0” (Repnikova and Fang 2018). A human touch may be the magic weapon to reset such a process when living in the era of codes.

## Footnotes

1 Weixin is the name of the Chinese version of WeChat. Weixin is available to PRC-registered users, while WeChat is available to users who register with non-PRC mobile phone numbers. The two versions are inter-operatable, particularly at the user end. The author of this article is a WeChat user; all others involved in the research are Weixin users. For simplicity, this article uses “WeChat” to refer to both Weixin and WeChat.

2 There are 34 administrative divisions in China, comprising of 23 provinces (including Taiwan, according to the official Chinese policy), five autonomous regions, four municipalities, and two special administrative regions (Hong Kong and Macau). There are 33 health codes based on administrative divisions accessible via WeChat—Taiwan is excluded. There are 34 health codes accessible via Alipay—Taiwan is included.

3 The interviews were conducted for a collaborative project on digital kinship care in China, with the ethics approval from the author’s home institution in early 2021. One China-based colleague conducted interviews in Wuhan. Later, a Sichuan-based research assistant was employed to conduct additional interviews in Chengdu. Both were asked to “walk through” the technical features of their health codes.

4 It is beyond the scope and space of this article to provide an analysis of China’s silver economy, which is growing with China becoming an aging society and rising proportion of elderly internet users. See the China Internet Report by SCMP Research (2021) and Mob Tech (2021)’s report on China’s silver economy for views from the industry.

5 Digital refugees, digital migrants, and digital natives can be roughly grouped as the demographic cohorts of Boomers (1940–59), Gen X (1960–79), and Gen Y and Gen Z (1980–2010).

6 Outside lockdown times, older people can normally get about without the need for health code checks to access shops and parks. Government-run services like banks, hospitals, and buses are normally stricter in requiring one to show their health code before entry.

7 Posted by a netizen nicknamed “Prince” on 15 May 2020 (https://bit.ly/39lMyAw). China’s Skynet system (tianwang xitong) is a nationwide big-data-driven video surveillance system. It is often regarded as equivalent to China’s social credit system. See, for example, Shen (2018) and Mozur (2018), for further details.
